# Learning from Routing Information for Detecting Routing Misbehavior in Ad Hoc Networks

**DOI:** 10.3390/s20216275

**Published:** 2020-11-04

**Authors:** Robert Basomingera, Young-June Choi

**Affiliations:** 1 Department of Computer Engineering, Ajou University, Suwon 16499, Korea; basorot@ajou.ac.kr; 2 Department of Software and Computer Engineering, Ajou University, Suwon 16499, Korea

**Keywords:** ad hoc network, attack detection, machine learning, data sharing algorithm

## Abstract

Owing to ad hoc wireless networks’ properties, the implementation of complex security systems with higher computing resources seems troublesome in most situations. Therefore, the usage of anomaly or intrusion detection systems has attracted considerable attention. The detection systems are implemented either as host-based, run by each node; or as cluster/network-based, run by cluster head. These two implementations exhibit benefits and drawbacks, such as when cluster-based is used alone, it faces maintaining protection when nodes delay to elect or replace a cluster head. Despite different heuristic approaches that have been proposed, there is still room for improvement. This work proposes a detection system that can run either as host- or as cluster-based to detect routing misbehavior attacks. The detection runs on a dataset built using the proposed routing-information-sharing algorithms. The detection system learns from shared routing information and uses supervised learning, when previous network status or an exploratory network is available, to train the model, or it uses unsupervised learning. The testbed is extended to evaluate the effects of mobility and network size. The simulation results show promising performance even against limiting factors.

## 1. Introduction

Ad hoc wireless networks are infrastructureless networks where devices collaborate to exchange data in a decentralized environment built or dismantled dynamically. Ad hoc networks are also known for their dynamic topology with restrained resources, which complicates the implementation of security solutions compared to networks with an infrastructure. Applications of ad hoc networks are in critical areas such as disaster relief, military activities, remote areas, vehicular ad hoc networks, and flying ad hoc networks [1,2,3,4]. Ad hoc networks have requirements to fulfill to become more reliable and realize all their potential applications. Among different requirements, ad hoc networks need to implement mechanisms to detect, prevent, and mitigate security issues because they are critical to mission-critical networks where a single failure leads to unwanted consequences. In general, wireless networks are vulnerable to attacks such as jamming or spoofing. It is worse in ad hoc networks due to their properties because their routing protocols were initially designed without considering security factors. Several studies on security mechanisms in wireless networks have dealt with different types of attacks; hence, this paper focuses on detecting routing misbehavior attacks in ad hoc wireless networks. Several techniques may be applied to achieve a detection system for routing misbehavior attacks. Such techniques include the use of threshold values, statistical analysis, finite state machine (FSM), rule- and signature-based schemes, machine learning (ML) models, and cryptography [5,6,7,8].

Each technique has its benefits and drawbacks; for example, cryptography solutions are challenged to ensure a stable key management system and cope with computing resources limitations, which may lead to using less reliable algorithms. The detection of unprecedented attack scenarios is a challenge for heuristic systems relying on threshold values, rules, signatures, or FSM methods, as they are designed to specifically overcome predefined scenarios, and their static nature means they require an update to adapt to new scenarios. To efficiently detect new attack scenarios, the use of ML algorithms has more promise. ML algorithms focus on building a system that continuously improves the performance based on prior results. Moreover, ML models can adapt to new paradigms in the network from collected information [7]. Hence, in this work, we opted for an ML-based detection owing to its potential to even detect unprecedented attack scenarios. Applying ML on detection systems requires serious majors and steps such as keeping the scope narrow and clearly understanding the threat model [9]. Therefore, this work only focuses on detecting attack scenarios that lead to routing misbehavior attacks targeting control packets in ad hoc wireless networks. The detection in wireless ad hoc networks can be performed by each host node or by a cluster head. Some works, such as in [10,11,12], proposed cluster-based schemes and improved the performance; although, the burden is placed on the cluster head. Cluster-based schemes have issues such as the following:

- The cluster head can only start the detection after it has been selected as the head.
- The hosts cannot be or would contend with a delay in being protected (by themselves or another cluster head) when the cluster head fails or moves away.

A host-based detection system, such as in [13,14,15,16], allows each node to handle its security. Unfortunately, it is not always efficient, as a single node might not have all the necessary information and the same detection is probably already being run by another node. To achieve a better performance in a network without infrastructure and with dynamic topology, it is better to design the detection system in a distributed or cooperative scheme [17]. This work proposes a detection system that can run either as host- or cluster-based and exploits both detection schemes’ advantages. The simulation results of the proposed system demonstrate a superior detection accuracy compared to other related systems. This paper builds from the previous work [18] and its contributions are summarized as follows:

- Proposes routing information-sharing algorithms in ad hoc networks.
- Compares the detection performance of different learning algorithms and proposes a detection scheme that uses the shared routing information for both host- and cluster-based detection.
- Analyze the memory and bandwidth overhead caused by the proposed routing information-sharing algorithms.

## 2. Routing Process and Attacks in Ad Hoc Networks

### 2.1. Route Cache (Routing Table)

**Figure 1:**
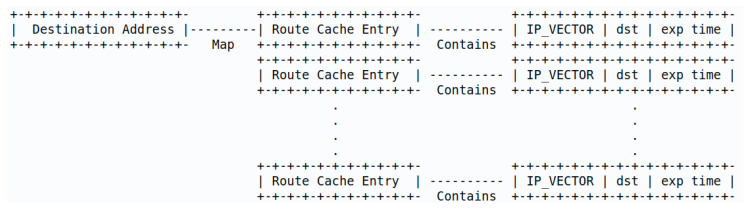
Route cache structure [24].

In ad hoc networks, a distributed routing protocol is used to discover the hop-to-hop route for data packets. This process requires collaboration between nodes by acting as endpoints and as intermediary routers. Their design philosophy classifies ad hoc routing protocols as table-driven (They maintain a list of all possible routes through periodical updates by nodes broadcasting their routing information throughout the network; hence, they are also called proactive), on-demand (They find routes by broadcasting route request packets into the network every time a data packet is to be sent; hence, they are also called reactive), and hybrid (They use a hierarchical architecture and create several groups in the network with a different role to each group) [19,20,21]. The mechanism that allows a node to discover a route (also referred to as a path or a link) to other nodes is performed by broadcasting packets with requests or unicasting packets announcing routes. These packets that allow nodes to discover or announce routes are called control packets. The working process of control packets slightly varies by the type of routing protocols. For example, in a reactive routing protocol, nodes identify routes by broadcasting request packets every time a data packet has to be transmitted. This route discovery process runs with two messages: route request—RREQ (the broadcasted packet by a node to identify a link to another node) and route reply—RREP (the reply sent to the originating RREQ). The resulting routing information is stored in a route cache. Hence, a route cache is storage space within a node that caches routing information collected by the node over time. Each node adds information to its route cache as it learns of new links with other nodes as well as when the node receives control packets (RREQ, RREQ, etc.). Nodes also update information in their route cache when they learn that a link is broken [22]. An example of a route cache structure for dynamic source routing (DSR) protocol is shown in Figure 1, where IP_VECTOR is the route vector with the IP addresses of intermediate nodes. For proactive protocols, the equivalent of a route cache is a routing table that is collected and stored periodically from the periodical updates performed in the topology discovery process. At the end of the topology discovery process (accomplished through advertised links, by using control packets), each node maintains a routing table full of routes to other nodes in the network [23]. The main difference between reactive and proactive is that the time routing information updates are conveyed.

### 2.2. Attack Model: Routing Misbehavior Attacks

Ad hoc wireless networks are particularly susceptible to network attacks, these are grouped into three categories based on the attacker’s target—data traffic attacks (targeting data packets exchanged between nodes), routing attacks (targeting routing process), and physical layer attacks (targeting data link layer and physical layer communication). Ad hoc networks share similarities with other wireless networks; meanwhile, some of the attacks encountered in other networks are also encountered in ad hoc, with some exceptions. For example, because the Internet of Things (IoT) extends its communication to the Internet, it inherits Internet-based attacks, which are not possible on an ad hoc network implemented among devices only [25,26]. During and after network initialization, nodes start the routing process to discover routes to reach their desired destination; hence, the first packets exchanged in the network are control packets. Some of the routing misbehavior attacks aim to abuse control packets. In this work, we focus on the detection of two attacks:

- Falsifying attack (or route falsification), malicious nodes broadcast falsified routes.
- Sybil attack (or identity spoofing/imitation), a node fakes the identities of other nodes or assigns itself multiple fake identities.

The falsifying and Sybil attacks, if successful, create malicious routes (We refer to the malicious route as a link that contains wrong or fake hops that were created on purpose by an attacker node) in the route cache of victims. They allow an attacker to act as the relay node or imitate the victims so that other nodes send the data packets to the attacker, believing that they are legitimate. The success of this attack results in more attacks. The Sybil and falsifying attacks are manifested in different scenarios, which is one of the reasons that the detection is trickier with a heuristic-based detection system. For example, falsifying attacks can happen as either of the following:

- The malicious nodes occasionally fake only a specific route and behave appropriately for others.
- The malicious nodes occasionally fake every single route for which they receive a request.
- The malicious nodes fake every possible route every time they have the possibility to attack.

For this work, the attack model scenarios are generalized to where the attacker nodes occasionally (to hide their malicious activities) run the attacks on the victim nodes as follows:

- The attacker node imitates the identity of the victim node and replies to requests while claiming to be the victim node (Sybil attack). The imitation is done by arbitrarily changing or spoofing its own host identity.
- The attacker node replies to requests from other nodes by falsely claiming that it directly links with the victim node (falsifying attack).

## 3. Related Works

Different types of security systems in ad hoc networks have been proposed to detect, prevent, or mitigate routing misbehavior attacks and their success-resulting attacks. Most of these systems are proposed as either cryptography-, rule-, signature-, trust-, or learning-based for detection and prevention. Other systems are proposed with the same techniques but rather to mitigate the attacks that result from successful routing misbehavior attacks such as blackhole, man-in-the-middle, or denial-of-service (DoS).

### 3.1. Cryptography Systems

Cryptography-based systems in ad hoc networks have mostly been proposed by redesigning routing protocols or modifying how routing information is shared. In studies like [27,28,29,30], they proposed schemes that added encryption and digital signatures in the routing process to secure dissemination of control and data packets. However, cryptographic operations are still considered as expensive on resource-constrained devices [31]; although, other works, such as [32,33], addressed issues of key generation, key distribution, and key management. Other works, such as [34], tried to address the issue of encryption complexity regarding the time taken for encryption and decryption.

### 3.2. Heuristic and Learning Systems

Learning-based detection systems learn behaviors of a network to identify later malicious activities based on the patterns learned that might facilitate routing misbehavior attacks. In [13], a decision-tree-based artificial immune system was used against the attacker node in a host-based detection design on mobile ad hoc networks (MANET). The work in [35] was based on routing information for the detection of a malicious node. It supervised and learned the sequence number of each RREP with training data updated dynamically at regular intervals for three features in a host-based detection design. In [36], they proposed a flow-based detection system that leverages ML and software-defined network in a tactical MANET. The system works in global and local controller design, gathering data from mobile nodes. Among these learning-based systems, the cluster-based is the only one to achieve performance above 90%. However, it is designed to run as a cluster-based-like scheme only. This design leaves room for improvement in designing systems that can adapt to both cluster- and host-based detection systems while keeping good performance.

In contrast, rule- and signature-based systems try to identify abnormalities by following a predefined set of patterns. Trust-based systems may be compared to rule-based systems as they create and analyze a set of information based on the provided or preassigned cognitive judgment that works as a trust evaluation system to decide trustworthy information of devices in the network. In [37], they proposed a biologically-inspired spider-monkey time synchronization technique to detect Sybil attack in large-scale vehicular networks. In [14], the authors proposed a detection scheme based on an analysis of the received signal strength as an indication to counter identity attacks. This analysis was possible when the receiver node knows the maximum speed a node could have in the network. The receiver node estimates the distance between itself and the sender. It compares if the change in the distance covered is less-than or equal-to the one that could be covered by the same node at its maximum speed. In [38], they proposed a Sybil attack detection method with power control that adopts the received signal strength as time series to compare similarity among all the received series’.

All of these prior related systems are implemented in different schemes and with different techniques. In most cases, the most efficient are resource exhausting, such as cryptography-based systems; while others use lightweight techniques, such as thresholds values, for handling attacks but mostly end up with less accuracy. In this work, we learn from all these systems and aim to design a lightweight, learning-based detection system that still offers acceptable accuracy despite being simple and able to be used as host-based or as cluster-based without further complex changes.

### 3.3. Aftermath Attacks Mitigation

Other works tried to mitigate attacks that resulted from successful routing misbehavior attacks, notably, DoS. In [16], SVM and threshold-based detection methods were proposed against DoS by relying on three features: the average number of packet drops, average packet reception, and average packet arrival interval. In [39], they suggested a secure multipath protocol. The protocol encodes messages into different pieces to tackle the issue of identifying the right route in a multipath network, hence avoiding the DoS. In [40], the authors proposed a reCAPTCHA controller mechanism that prevents automated DoS attacks that may result from attackers making the desired destination unavailable.

## 4. System Design

**Figure 2:**
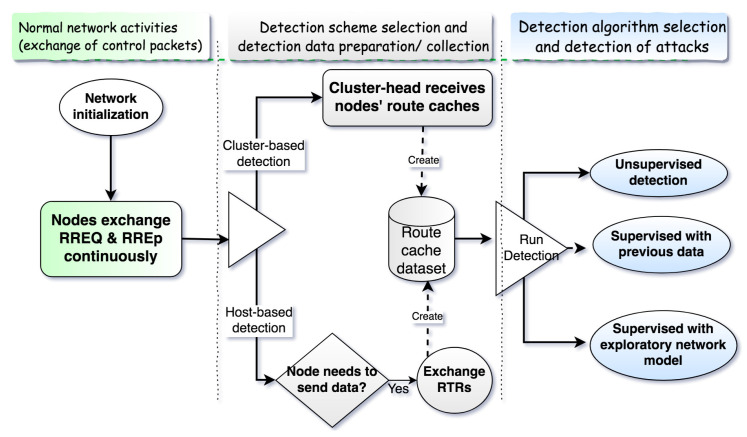
Detection process overview. RTR—request-to-receive.

The proposed detection system has two functioning schemes: host- and cluster-based. A host-based detection scheme is when a node runs the detection system itself. A cluster-based detection scheme is when the cluster head runs the detection for its cluster. Both schemes run the detection on a dataset created cooperatively by several nodes using proposed algorithms that allow the sharing of routing information. The algorithm design is guided and constrained by the nature of the network. Figure 2 shows an overview of the detection process, which is made up of three steps after nodes are initialized and continuously exchange control packets. The steps are as follows:

- *Detection scheme selection*: The nodes in the network decide among host-based and cluster-based detection. Cluster-based detection is used when the cluster head is available and has enough resources.
- *Route caches collection and processing*: Nodes use routing-information-sharing algorithms to send their route caches to the cluster head (cluster-based detection) or exchange among neighbors (host-based detection).
- *Algorithm selection and attack detection*: The detection algorithm is decided among supervised learning (when the training dataset is available) and unsupervised learning (when the training dataset is unavailable). The dataset of route caches is used by the detection algorithm, which identifies malicious routes.

### 4.1. Routing-Information-Sharing

Routing information-sharing approaches have been studied in other types of networks with a different perspective. For example, the work in [41] uses shared information to perform mapping of a wireless personal area network (where mobility has no significant impact) in a directed acyclic graph. The mapping was placed at the border router. The border router is a benefit that is not available in an ad hoc wireless network, as there is no central device that is granted to be accessible all the time. In this work, we designed route cache sharing algorithms used to share routing information for both cluster- and host-based detection. The routing-information-sharing objective is not to replace the usual routing process. Instead, it is to allow nodes to create a dataset used to detect malicious routes within the route caches. The usual routing process remains functional and vital in discovering links between nodes.

#### 4.1.1. Neighbors Route Cache Sharing Algorithm

**Figure 3:**
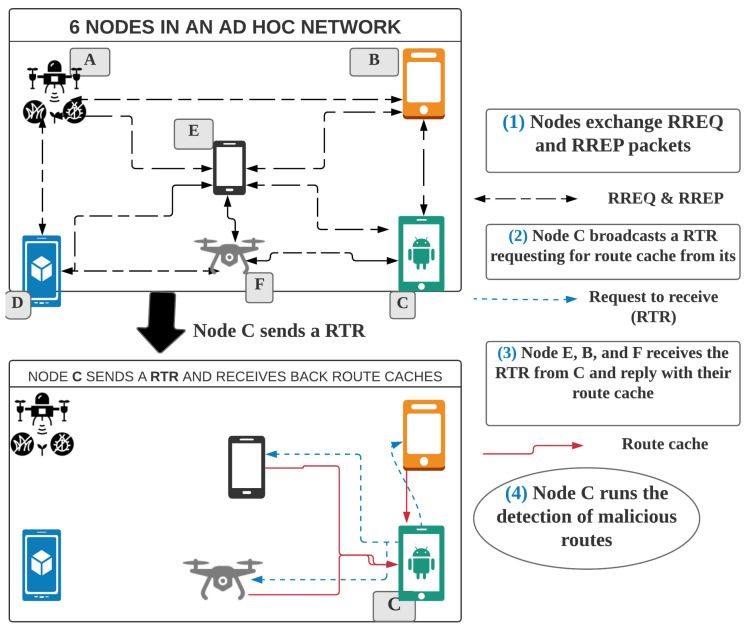
Cooperative host-based detection. RREQ—route request, RREP—route reply.

The route cache sharing algorithm for neighbors is a routing-information-sharing algorithm that allows nodes to share their route caches with their one-hop neighbors. At the end of the sharing process, a node has a copy of its neighbors’ route caches. As shown in Algorithm 1 and illustrated in Figure 3, the algorithm runs on every node, but cooperatively with its neighbors, as follows:

- Whenever a node wants to send a data packet and its route cache has changed above a preset threshold (The threshold of changes should be considered before sharing the route cache. It can be agreed among nodes or set by each node individually. In our simulation, we use a 10% threshold.) since the last detection, it broadcasts a request-to-receive (RTR) (A UDP/IP signal packet is broadcasted by the node that wants to receive the route cache data from its neighbors. RTR is received and read by every node in the transmission range of the sender node (one-hop node.) packet (In Figure 3, node C broadcasts the request) with a time to live (TTL).
- The neighboring nodes receive the broadcasted request and reply (In Figure 3, nodes B, E, and F reply to C) with their route cache to the requester node.
- When the requester node receives responses from all its already known neighbors, it combines all received route caches and creates a dataset. Otherwise, if the TTL is passed before all responses are received, the requester node uses only the received route caches.

Each node maintains a route cache dataset (A collection of route cache collected from other nodes.) in its storage, and this dataset is not destroyed after the detection. It is retained, and once the node receives an update from its neighbors, it updates the content. The node removes the route cache from a specific node when it learns that its link is dead.
**Algorithm 1:** Neighbors Route Cache Sharing
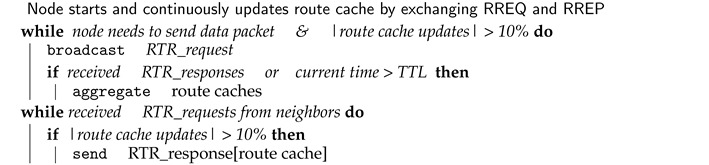


#### 4.1.2. Cluster Route Cache Sharing Algorithm

In a cluster-based system, nodes rely on a single node—the cluster head—for their security. This methodology is useful when a single node still has adequate resources, while others are exhausted. However, it also results in more resources being consumed by that single node. For this work, the scope of routing-information-sharing is limited to when the cluster head is already selected. Other works, such as [42,43,44,45], are a good source for algorithms and analysis on the cluster head selection process. For this scheme, we have three main assumptions about the clustered network:

- It uses the spanning-tree mechanism, as discussed in [46], to avoid endless forwarding.
- It uses adaptive relay selection, as discussed in [47], to build a proper network relay selection.
- It uses the concurrent control of mobility and communication, as discussed in [48], to ensure concurrent controls where needed.

The algorithm shown in Algorithm 2 shows that all nodes send their route caches to the cluster head periodically if there have been changes in their route caches since the last detection or as per request from the cluster head. The periodical interval T is decided based on average speed of nodes *S* and their transmission range τ as T=S/τ.
**Algorithm 2:** Cluster Route Cache Sharing
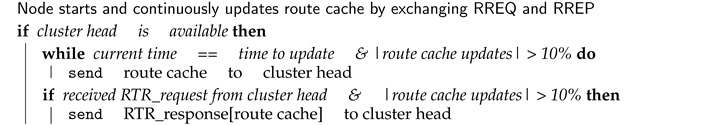


#### 4.1.3. Potential Attacks on Sharing Algorithms

With these proposed sharing algorithms, malicious nodes may attempt to take advantage of them to initiate other attacks such as DoS. Malicious nodes may also cause other nodes to use their resources unnecessarily exhaustively. During the design of the algorithms, specific design decisions were considered to counter those potential attacks while avoiding heavy computations:

*One-hop-sharing (for host-based):* the reason for using one-hop sharing of the route cache is to avoid creating massive communication and memory overheads in the network. This decision is acceptable because it is the neighboring nodes that have similarities in routing information. The routing process itself remains functioning normally, and only route cache sharing is limited to one-hop-sharing.

*A threshold of changes:* a malicious node may spread fake routes to create changes in the route caches; hence, unnecessarily triggering the detection process. This issue is avoided by choosing a threshold for changes required before activating the detection. This decision allows nodes to avoid being victims of the route cache sharing algorithms itself. Whenever a node receives an RTR-request, it only replies to it if its route cache has been updated above the preset threshold.

*Detection when needed:* a node runs the detection system only when it has data packets to be sent, and its route cache has changed. To avoid that, a node exhausts the resources quickly and unnecessarily.

### 4.2. Detection Model

After receiving route caches, nodes can then run the detection of malicious routes using an ML algorithm. ML algorithms are either supervised, hybrid, or unsupervised. In this work, we only considered “unsupervised” and “supervised” for the entire RREQ timeline (Route cache data for each node are updated every time there is a new RREQ (which results in RREP exchanged); meanwhile, one RREQ sent out represents a new network state, not because the RREQ changes the network, but because it allows gathering the changes that happened in the network. An RREQ timeline is the entire set of RREQ that happened throughout the network time.). Both unsupervised and supervised use the same dataset, with one main difference being that for supervised, route caches are labeled. In contrast, for unsupervised, there are no labels. We tested supervised in two variants: supervised with previous data and supervised with exploratory network.

#### 4.2.1. Supervised Detection with Previous Data

Previous route cache data are labeled and used to train the model. Meanwhile, another party or process with knowledge of the network’s previous status is used to label the data. These labeled routes can then be used for training the model. This method is more suitable to use when the previous behaviors of the network are known. In the simulation, we explored this method of labeling in the following sequence:

- At RREQ timeline of $T_{i}$, collect the route cache dataset. The collected dataset is labeled based on prior knowledge of the network and the model is trained with the labeled routes.
- At RREQ timeline of $T_{n}$ (n>i), run the detection on dataset collected at $T_{n}$ by using the model trained at $T_{i}$. In simulation, we use n=i+10 for cluster-based and n=i+5 for host-based.

#### 4.2.2. Supervised Detection with Exploratory Network

This method is about creating a network with parameters such as network size, maximum speed, and mobility strategy, similar to the network to be tested. Once the training network is set, the network behaviors are observed, and hence the routes are labeled and used to train the model. The model is deployed on the actual network for the detection of attacks. This method is more suitable to use when some information (network size, mobility strategy, etc.) about the network to be monitored can be anticipated or is known.

#### 4.2.3. Unsupervised Detection

When using the unsupervised model, there is no need for labeling the routes, as the model itself identifies the abnormalities in the route cache by looking for outliers. This method is more suitable to use when the network’s previous behaviors are not known. The operational flow is that, after nodes are initialized, they run the route cache sharing algorithm to create a dataset. Then, the unsupervised model is used to identify malicious routes by looking for outliers.

#### 4.2.4. Supervised Detection Algorithm

To decide which algorithm to use in the supervised learning, we compared four algorithms: k-nearest neighbor (KNN), support vector machine (SVM), decision tree (DT), and Long Short Term Memory—Recurrent Neural Network (LSTM-RNN). Each is selected for the following reasons:

- KNN uses a method for discriminating corresponding data based on N number of training data located in a specific space while using every feature of similarity for processing their similitude so that it is good for classification tasks [49,50,51]. KNN has several variants to improve its efficiency, such as for fast retrieval and deciding neighbors [52,53], or for dealing with real-time prediction issues in resource-scarce devices [54]. We implemented KNN with the following parameters: n_neighbors = 1, leaf_size = 1, algorithm = auto, and weights = uniform.
- SVM uses a nonlinear classification with different kernel functions, allowing functional generalization abilities for binary classification [55,56]. We implemented SVM as a support vector classifier (SVC) with the following parameters: C = 1, gamma =0.1, SVC kernel type is radial basis function of exp(γ$||x-x^{\prime }\left|\right|\gamma^{2}$), Decision_function_shape=ovr, and Tol = 0.001.
- DT is known for its performance at mapping nonlinear-relationships and hence, solving the corresponding classification tasks. Owing to routing information’s spatial nature, DT, when adequately configured, may be the most suitable classifier to identify abnormalities in the route cache datasets. DT variants improve the performance in ways such as using the gradient-boosted DT to solve high-dimensional sparse output [57], or address issues of adversarial attacks on DT algorithms [58]. We implemented DT with parameters: criterion = gini, and max_depth = None.
- RNN has cyclic connections that make it compelling for modeling sequences [59]; hence, we used it with LSTM, where hidden layers of RNN are replaced with LSTM cells. We implemented LSTM-RNN with four layers: initial Embedding layer, two middle LSTM layers, and Dense layer. The layers are implemented with dropouts (to handle overfitting) by using Keras library with *TensorFlow* [60] backend.

For each algorithm, parameters were calibrated by a grid search approach. The features used for detection are addresses of the source and destination nodes, addresses of intermediate nodes, and total number of hops (the size of IP_VECTOR in the route cache). After a series of trials, these features were selected, as they showed better performance than other combinations of the route cache elements. Among other combinations that were checked against was to add the route expiration time and to remove the route length. In Section 5, we select one model based on the simulation results.

## 5. Simulation Setup and Results

### 5.1. Simulation Environment

As a simulation tool, *ns-3* [61] is used to simulate a DSR network. Some simulation parameters are listed in Table 1. For the mobility of the network, nodes followed a randomly uniform distributed speed, with the minimum speed being 0 m/s and the maximum being one of 1.38 m/s (average speed of a pedestrian), 4.3 m/s (average speed of a cyclist), 11.11 m/s (average speed of a horse), and 20 m/s (the maximum speed for a *DJI Phantom 4 PRO* drone in S-mode). The number of malicious nodes μ is arbitrarily set proportionally to the total number of nodes N as μ=⌈N/10⌉. To analyze the performance of the detection system, we use five metrics that allow us to evaluate the effectiveness on the performance of a machine learning model as follows:

- True Positive Rate (TPR): This shows the proportion of routes that are predicted to be malicious and are actually malicious. It can also be referred to as the sensitivity of the model.
- False positive rate (FPR): This shows the proportion of routes that are predicted as malicious but are not genuinely malicious.
- Accuracy: This is the overall correctness of the classifier. It shows the ratio of correct predictions (True Positive—TP and True Negative—TN) against the total number of predictions (TP, TN, False Positive—FP, and False Negative—FN). It shows the ratio of correctly identified routes as either being normal or being malicious. It is calculated as shown in (1),
  (1) $$Accuracy=\frac{TP+TN}{TP+TN+FP+FN}.$$
- F-score: the harmonic weighted average of precision and recall, which is an important metric—better than accuracy—in case where the distribution of classes in the dataset is imbalanced. It is calculated by (2)
  (2) $$F-score=\frac{2\cdot TP}{(2\cdot TP)+FP+FN}.$$
- Area under the curve of the receiver operating characteristic (AUROC): This represents the performance averaged over all possible cost ratios. Meanwhile, it shows the averaged TP against FP for various threshold values.

As they show performance metrics of the model from several perspectives, these metrics are enough to understand whether the classifier is effective in identifying malicious routes, although they do not make further analyses such as the resource consumed or training time.

### 5.2. Supervised Model Selection

**Figure 4:**
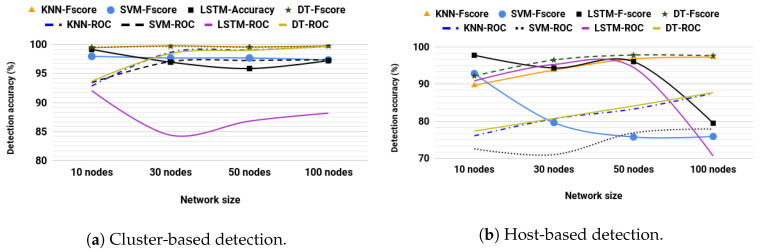
Performance comparison of classification models. KNN—k-nearest neighbor, SVM—support vector machine, LSTM—long short term memory, DT—decision tree, ROC—receiver operating characteristics.

The F-score and AUROC were analyzed for SVM, KNN, LSTM, and DT on both host- and cluster-based schemes. We performed simulation analysis for a network with nodes moving at a maximum speed of 4.3 m/s. For cluster-based detection, the results in Figure 4a show that DT demonstrates marginally superior performance over KNN. At the same time, SVM and LSTM-RNN lag behind, especially as network size increases. LSTM-RNN shows the worst AUROC performance. By definition, AUROC is the trade-off between TPR and FPR across different thresholds, which shows a statistical summary for the predictor’s correctness [62,63]. Hence, from LSTM-RNN’s AUROC results, we can infer that LSTM-RNN produced a disproportionate trade-off between TPR and FPR. An extended analysis could precisely explain the reason for such a result. However, intuitively two details can explain why. These details are the nature of routing information and the working scheme of RNN.

RNN uses recurrent cyclic connections to model sequence between elements, which means layers recurrently exchange their weights and bias. The fact that routing information has multiple redundant or comparable links may result in several normal routes being confused as abnormal by the model. However, note that LSTM-RNN’s AUROC remains above 0.8, which is a good result.

Using host-based detection, the results illustrated in Figure 4b are the average of all nodes in the network. The results show DT as the best detector with a slight margin over KNN. Both DT and KNN improve their performance as the number of nodes increases, while SVM and LSTM-RNN are affected. The performance of LSTM-RNN stays steady until a 50-node network. For a 100-node network, the performance significantly drops. Individual analysis for each node’s performance shows a higher ratio of nodes with much lower performance in a network of 100 nodes than in other ones, which explains the sudden drop. This can be observed as the randomness of the network activities affects the LSTM-RNN more on a giant network than it does on other models. Based on the results in Figure 4a,b, we choose the DT algorithm for supervised learning. For the remainder of this paper, supervised learning is used with DT.

### 5.3. Simulation Results

#### 5.3.1. Supervised Detection with Previous Data

For the host-based detection with route cache sharing algorithm for neighbors, our simulation is repeated three times. We analyze the detection performance pertaining to the network size change and nodes’ mobility. The detection results using DT, as listed in Table 2 and Table 3, show that the average detection accuracy is 96%.

It is observed that higher mobility hurts the performance of the detection system. This mobility effect is because when nodes are moving faster, they create several changes in the route cache. The changes may take time to be reflected in the dataset of neighboring route caches. Moreover, the results do not show monotonically increasing performance when the network size increases because, in simulation parameters, the number of malicious nodes increases with the increase of network size. For the cluster-based detection, we simulated a single cluster network and repeated each simulation three times to observe the performance while analyzing the detection performance pertaining to network size and nodes’ mobility. In cluster-based detection, the model training time is, on average, 7.25 ms for 10-node network, 9.82 ms for 30-node network, 16.90 ms for 50-node network, and 46.87 ms for 100-node network.

The detection results by DT, as listed in Table 4 and Table 5, show that the accuracy averages 98%. The results in Table 4 show that the increase of network size helps the classifier overcome the randomness and higher mobility of nodes. Table 5 shows that mobility does not have a more significant impact in the cluster as it does in the host-based detection. The reason for such results is that even if mobility is higher, the fact that the cluster head collects data from several nodes gives it a chance to overcome frequent changes in the network.

The effect of network size or mobility, individually, does not manifest a higher impact in cluster-based detection. However, a combination of both shows a significant impact; for example, the accuracy of a 30-node network moving at 20 m/s is 89.5% while it is 98.5% for a 50-node network moving at 4.3 m/s. A key observation is that cluster-based detection has a higher and more stable average accuracy than host-based detection, presenting a more fluctuating performance. The reason is from the nature of the mobile ad hoc network and parameters used in the simulation. Nodes in the simulated mobile ad hoc network have random waypoint mobility and randomly change their speed, resulting in random network behavior. The randomness of the network has a significant impact on routing information, which, unfortunately, affects the host-based detection more than cluster-based detection. The cluster-based detection uses data from several nodes, which allows it to overcome the network’s randomness, while host-based keeps fluctuating due to randomness impact.

#### 5.3.2. Supervised Detection with Exploratory Network

**Figure 5:**
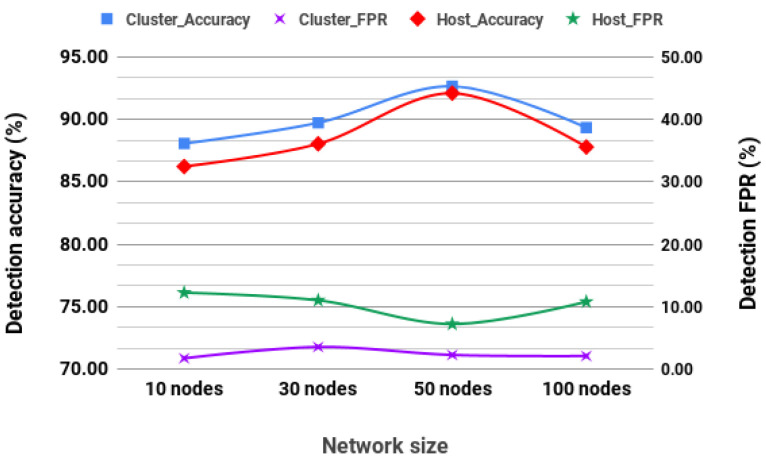
DT Exploratory network detection (Max speed of 4.3 m/s).

A test network was set at a speed of 4.3 m/s, and the network behaviors are logged to identify malicious routes. These logs are then used to label the dataset in order to train and cross-validate the detection model, which is only trained on the dataset from all nodes (as in cluster-based detection). After the model is trained, another network is simulated with 4.3 m/s nodes’ average speed. The trained model is deployed to the network and is run as both a cluster-based and host-based detection system. The results in Figure 5 show that nodes achieve a performance above 85%. Performance from the supervised detection with an exploratory network improves gradually as the network size increases to a 50-node network, which seems to be the peak, as the performance starts to decrease as the network size increases.

#### 5.3.3. Unsupervised Detection

One of the disadvantages of supervised learning is that it is not always possible to obtain a proper training dataset or abide by the training time. Nevertheless, the model’s accuracy depends on the training dataset’s correctness [8,56]. In some instances, this issue can be surmounted using unsupervised learning, where models are permitted to discover patterns in the data without being assigned labels. For cluster-based detection, we use covariance module’s *EllipticEnvelope*. The *EllipticEnvelope* uses a covariance estimate to detect outliers in a Gaussian multivariate dataset [64]. We implemented *EllipticEnvelope* with the following parameters: assume_centered = True, contamination = 0.15, random_state = rng, store_precision = True, and support_fraction = None. For host-based detection, we use *One-class SVM*, which is a kernel-based learning algorithm of multiple parallels separating hyperplanes in a reproducing kernel Hilbert space [65]. We implemented *One-class SVM* with the following parameters: cache_size = 200, coef0 = 0.0, degree = 3, gamma = 0.001, kernel = rbf, max_iter = −1, nu = 0.15, shrinking = True, tol = 0.001, and verbose = False.

**Figure 6:**
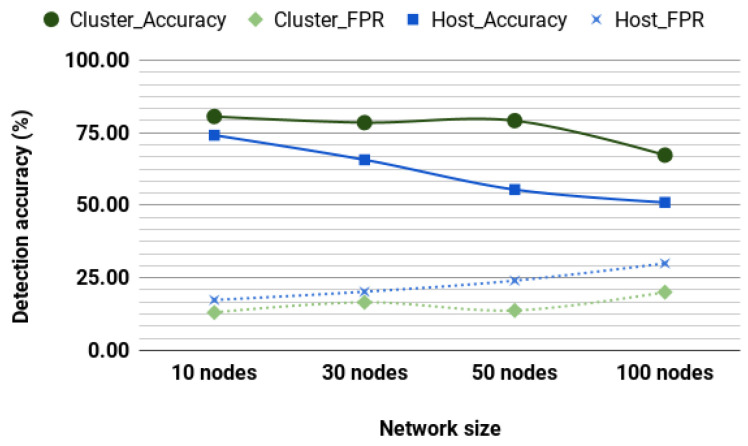
EllipticEnvelope and One-class SVM unsupervised detection (Max speed of 4.3 m/s).

These algorithms were selected owing to their performance against other unsupervised algorithms. We conducted simulation on a network of nodes moving at 4.3 m/s. Figure 6 shows the detection accuracy and FPR for a cluster-based detection system and a host-based detection system. The performance of unsupervised learning is affected as network size increases. This decrease may be doomed to the way unsupervised models classify by identifying outliers. In routing information, outliers may mean links with so many or very few hops, or links between nonclose nodes. Such outliers are more likely to be encountered in a giant network than in a smaller network, which is an advantage and a challenge as the model is likely to make more false classifications. When compared to the performance of the unsupervised models, supervised performed better. However, the unsupervised model still holds the advantage that they do not require training datasets.

## 6. Detection Analysis

The simulation results in Section 5 were run with assumptions that all nodes share their route caches, including the malicious nodes, and that all packets carrying route caches are successfully delivered to their destination. Unfortunately, these assumptions cannot always be valid. We then ran more simulations to analyze the detection performance with limiting factors and compared it against other detection systems.

### 6.1. Limiting Factors Performance

#### 6.1.1. Increase in Malicious Nodes

We ran the simulation three times for a network of 50 nodes moving at 11.11 m/s to analyze the performance when the number of malicious nodes changes. The analysis was performed considering cases when malicious nodes took over 2%, 10%, 20%, and 40% of all nodes. The accuracy is observed as 99.69%, 98.84%, 98.26%, and 97.75%, respectively. The FPR is also observed as 0.22%, 0.53%, 0.9%, and 1.49% respectively. Overall, the performance slightly deteriorates with the increase of malicious nodes in the network. The main reason for this deterioration owes to the fact that when there are many malicious nodes in a network, they create a bias in the route cache so that some fake routes may seem authentic. However, it does not show significant deterioration, which may be attributed to the fact that the attacks are not coordinated.

#### 6.1.2. Uncooperative Malicious Nodes

Malicious nodes would prefer not to collaborate with other nodes for their detection. Hence, typical scenarios are either of the following:

- Malicious nodes manipulate their route cache before sharing it with other nodes.
- Malicious nodes do not share their route cache.

When malicious nodes do not share their route cache data, the simulation results in Table 6 are shown to be less affected. The accuracy is comparable with the cases where route caches from malicious nodes are shared. This is possible because, in most cases, routes within malicious nodes are also present in the route caches of the neighboring nodes. However, a route is not checked if only the malicious nodes hold it.

#### 6.1.3. Packet Loss Effect

Mobility and repeated communication between nodes generate more packet losses in ad hoc networks [66]. We modeled the packet loss as a probability problem and observed the performance against the packet loss.

As illustrated in Table 7, the performance is affected by packet loss as the detection TPR decreases by approximately 7–10% when the packet loss probability increases by 40%.

### 6.2. Comparison of Detection Schemes

**Figure 7:**
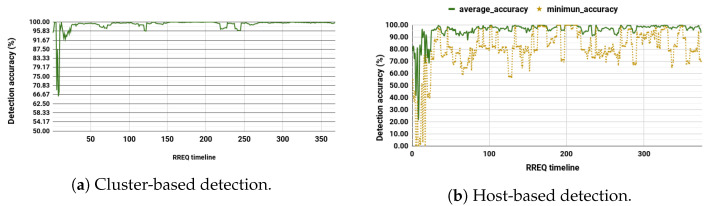
Cluster-based detection per RREQ timeline (Max speed of 4.3 m/s and Network of 50 nodes).

We performed a comparison to evaluate the change of detection accuracy over the RREQ timeline for both schemes. During the cluster-based detection, as shown in Figure 7a, the accuracy is maintained at an average of over 96% almost for the entire time.

For the host-based detection, Figure 7b shows that although the average accuracy remains higher than 95% in most cases, at some point in time, the minimum accuracy drops significantly for particular nodes. The drastic drop in the detection performance for a given node is blamed on the node’s movement. This implies that if a node moves farther from other nodes, it would not have sufficient data.

As ad hoc networks have limited resources, both host- and cluster-based systems should not be run simultaneously. Instead, they can be shuffled based on the situation of the node itself and the network; in general, this is to save resources and improve performance. Whenever the cluster head is unavailable, a node may (re)activate the host-based detection. The shuffling of systems would allow a node to achieve a better performance than when it would depend on host-based or cluster-based lonely detection. This is because, by having both systems, the node can use host-based detection when no cluster head is available. The node can also rely on cluster-based detection when it does not have enough data or resources. Table 8 summarizes the comparison with other detection systems and shows that the proposed scheme provides better detection performance.

### 6.3. Routing-Information-Sharing Overhead Analysis

#### 6.3.1. Routing-Information-Sharing Memory Overhead

The number of routes generated during the network lifetime depends on factors such as the number of nodes in the network and number of interactions that happen among nodes. After several simulations, by observing the change in terms of number of routes, we estimate the average number of total routes θ by Equation (3) (Equation (3) is tied to the parameters used in our simulation (Table 1). More studies are needed to determine a more general equation for different routing protocols and other mobility schemes. Here, *e* is the natural number (2.718) known as Euler’s number),

(3) $$\theta =\frac{\left(\mu +1\right)\cdot e\cdot N^{2}\cdot S}{\tau },$$

where the number of routes increases exponentially with the total number of nodes *N*. This value increases when the network contains malicious nodes μ. The speed *S* of nodes and their transmission range τ are both associated with the area size within which nodes are moving. Hence, if nodes are moving within an area that is not too big, the speed *S* would help increase the number of created routes because the nodes are moving faster around the same area. The transmission range τ inversely impacts the number of routes created. This impact is because, as nodes can transmit further, they can reach each other without creating more routes with intermediate hops.

From (3), if the total number of routes is θ, then on average, every node produces $\theta_{i}$ routes as follows:

(4) $$\theta_{i}=\frac{\theta }{N}.$$

The maximum number of neighbors that a single node can have is N−1; hence, the average maximum number of routes from other nodes that a node would have is given by (5):

(5) $$\theta_{i}\cdot \left(N-1\right)=\frac{(\mu +1)\cdot e\cdot N\cdot S}{\tau }\cdot \left(N-1\right).$$

Suppose *m* is the average memory size for a single route, the total required memory $\Omega_{h}$ in a host-based detection is expressed by (6):

(6) $$\Omega_{h}=\sum_{i=1}^{N-1}\theta_{i}\cdot m,$$

(7) $$\Omega_{h}=\frac{(\mu +1)\cdot e\cdot N\cdot S}{\tau }\cdot \left(N-1\right)\cdot m.$$

In the cluster-based detection, the cluster head receives, on average, a total of θ routes, which creates a memory overhead $\Omega_{c}$ as described in (8):

(8) $$\Omega_{c}=\theta \cdot m.$$

The results shown in Table 9—where $\Omega_{c}$ and $\Omega_{c}^{{}^{\prime }}$ (both in KB), respectively—shows the theoretical and simulation memory overhead in cluster-based detection. As the size of a single route *m* changes based on the number of hops, we obtained theoretical results with two arbitrary values—10 bytes and 75 bytes—and, in almost all cases, the simulation value is between the two theoretical estimated memory sizes. It is worth noting that this overhead memory analysis is limited to routing-information-sharing activities. It does not include other activities such as cluster head selection. One of the observations is that more malicious nodes within a network result in a higher memory overhead; meanwhile, malicious nodes create adverse effects on the network in different ways. Table 9 also shows host-based memory overhead with $\Omega_{h}$ and ${\tilde{\Omega }}_{h}$ (both in KB), respectively, showing the average theoretical memory overhead and the simulation median value for the host-based detection.

#### 6.3.2. Routing-Information-Sharing Bandwidth Overhead

The generated additional bandwidth overhead ⌀ due to routing-information-sharing is calculated by the number of packets propagated in the network Ψ due to the detection system multiplied by memory size of each packet. This number of packets propagated Ψ is approximately the same number of packets generated by a reactive routing protocol during the route discovery process. Reactive routing protocols send RREQ packets whenever a node needs to send a data packet and receive RREQ packets whenever other nodes need to send a data packet. Therefore, we can infer that the number of packets generated by the proposed detection scheme is less than or nearly the same (owing to sharing controlled by the *threshold change*) as the number of packets generated during route discovery of a reactive routing protocol. The number of packets propagated Ψ is calculated by expression (9)

(9) Ψ=|RTR_requests|+|RTR_responses|,

which is the sum of RTR-requests sent by the node and the RTR-responses received by the node from its neighbors.The total number of RTR-request packets generated by a single node is relatively equal to the number of data packets that are to be sent by the node. This is because nodes only send RTR-requests when they have data packets to send.

A single RTR-request has an IP header containing the source node address, the RTR id (2 bytes), the timestamp (4 bytes), and a TTL (1 byte); hence, the memory size of a single RTR-request Γ is 11 bytes in case of IPv4 and is generally expressed by (10)

(10) Γ=|src_IP|+|RTR_id|+timestamp+TTL.

A single RTR-response has the IP header with the source and destination addresses, the RTR id (to specify the RTR being responded to), and its route cache attached. The maximum number of routes that a single node is likely to have in its route cache is the number of routes, estimated by (3), from which the average can be inferred by (4). By assuming the size of one route as *m*, the total size of a single RTR-response k is calculated by (11)

(11) $$k=|src\_IP|+|dst\_IP|+|RTR\_id|+(\theta_{i}\cdot m),$$

(12) $$⌀=\sum_{i=1}^{\Psi }\Gamma_{i}+k_{i},\Rightarrow ⌀=\sum_{i=1}^{\Psi }21+\left(\theta_{i}\cdot m\right).$$

## 7. Conclusions

This work proposes host- and cluster-based detection systems, which run on route caches to detect malicious routes that resulted from routing misbehavior attacks targeting control packets. The detection system can be supervised (with labeled data or with exploratory network) or unsupervised. The average detection accuracy is around 97%, 90%, and 70%, respectively. Although the three approaches provide different performance, their usage cannot be decided based on their performance only. In contrast, the choice of usage should be relatively decided based on the situation of the network. Supervised with labeled data may be used if the network’s previous state is known to produce a training dataset. Such a scenario is likely when the network lasts longer and may be monitored. Supervised with an exploratory network may be used when some network details are known or may be anticipated. Unsupervised is usable when the network is entirely unpredictable, unmonitored, or short-lived. The proposed schemes’ complexity is relatively light as the two of the three proposed approaches do not require to train the model on the actual test network. Unsupervised learning does not require training and supervised with an exploratory network trains outside of the actual test network. The third approach, supervised with labeled data, does training in less than a second, mainly because the dataset size is relatively small. The simulation used DSR reactive routing protocol, but it sheds light on designing a more general approach to adapt to other routing protocols.

The proposed cluster-based detection is limited to the detection system, only after the cluster head is selected. The proposed detection scheme is designed to be applicable for a cluster head selected by any method. However, it is worth noting that the cluster head selection process creates problems in the network. The process of selecting the cluster head generates additional overhead (which can be huge if done extensively on a giant network) to the network. Hence, the future extended work will examine the impact of cluster head selection overhead on the detection system, quantify energy and computation resources by the detection scheme. An analysis of additional network metrics such as throughput, delays, or latency will also allow applying the systems to trending applications of ad hoc networks such as vehicular networks, flying networks, and even on IoT-based networks.

## Figures and Tables

**Table 1:** Simulation parameters. DSR—dynamic source routing.

Parameters	Value/Option
Simulation time	1000 s
Number of nodes	10, 30, 50, and 100
Simulation area	1500 × 1500
Transmission range	200 m
Mobility	Random waypoint
Routing/Mac protocol	DSR/IEEE 802.11b

**Table 2:** Host-based detection performance per network mobility (Network of 50 nodes). AUROC—area under the curve of the receiver operating characteristic, TPR—true positive rate, FPR—false positive rate.

Speed	Accuracy	F-Score	AUROC	TPR	FPR
1.38 m/s	97.488	98.319	93.040	87.230	1.195
4.3 m/s	98.083	98.756	89.837	80.779	1.503
11 m/s	95.743	97.050	87.829	77.452	2.670
20 m/s	94.381	96.056	85.897	74.290	3.461

**Table 3:** Host-based detection performance per network size. (Max speed of 4.3 m/s).

Network Size	Accuracy	F-Score	AUROC	TPR	FPR
10 nodes	92.218	95.315	83.705	72.677	9.876
30 nodes	97.085	98.127	89.249	79.941	2.246
50 nodes	98.083	98.756	89.837	80.779	1.503
100 nodes	97.805	98.424	92.534	85.774	0.610

**Table 4:** Cluster-based detection performance per network size (Max speed of 20 m/s).

Network Size	Accuracy	F-Score	AUROC	TPR	FPR
10 nodes	87.161	92.094	89.070	89.923	11.784
30 nodes	89.517	90.991	87.825	77.751	2.102
50 nodes	98.021	98.302	95.429	91.495	0.637
100 nodes	99.489	98.905	98.280	96.835	0.276

**Table 5:** Cluster-based detection per network mobility (Network of 50 nodes).

Speed	Accuracy	F-Score	AUROC	TPR	FPR
1.38 m/s	97.169	97.135	94.367	90.973	2.239
4.3 m/s	98.556	98.180	94.292	89.822	1.237
11.11 m/s	98.844	98.760	95.839	92.210	0.533
20 m/s	98.021	98.302	95.429	91.495	0.637

**Table 6:** Cluster-based detection performance without route cache from the malicious node.

Network Size	Accuracy	F-Score	AUROC	TPR	FPR
10 nodes	94.731	96.899	93.102	90.000	3.796
30 nodes	92.845	93.549	86.857	74.662	0.948
50 nodes	98.937	98.849	83.521	67.246	0.203
100 nodes	99.751	98.989	90.707	81.530	0.116

**Table 7:** Cluster-based detection against packet loss.

Loss Probability	Accuracy	TPR	FPR
2%	95.676	85.174	1.368
20%	95.413	81.904	1.982
40%	94.593	78.498	2.489
50%	93.345	76.118	2.807

**Table 8:** A comparison of the proposed system with others.

Related Works	Methodologies	Detection Techniques	Performance
M. Poongodi et al. [11]	cluster	A Trust Evaluation Mechanism with Clustering	95.80%
S. Zwane et al. [36]	local controllers	Adaboost with Packet- and Flow-based Detection	90.3–99.15%
D. Kosmanos et al. [67]	platoon of 4 vehicles	Random Forest, k-NN, and One-Class SVM with Data Fusion Techniques	72–99%
L. Jim et al. [13]	host	DT with Fuzzy Logic Block	~80% of PDR
M. Faisal et al. [14]	host	Threshold + Statistical Analysis	±93%
S. Abbas et al. [15]	host	Statistical Significant Testing	+90%
B. Subba et al. [12]	cluster	Threshold-based + Bayesian Game	81.33–91.78%
J. Cucurull et al. [68]	host	Statistical Anomaly Detector + Threshold	79%,95%
M. Alikhany et al. [69]	cluster	Weighted Fixed Width Clustering	92.34%
S. Kurosawa et al. [35]	host	Threshold + Dynamic Training	70–83%
Proposed scheme	host and cluster	Labeled Supervised with Route Cache Sharing	±96–±98%
Proposed scheme	host and cluster	Exploratory Supervised with Route Cache Sharing	±87–±93%
Proposed scheme	host and cluster	Unsupervised with Route Cache Sharing	±60–±80%

**Table 9:** Memory overhead.

*N* (μ)	$\Omega_{c}$(10)	$\Omega_{c}$(75)	$\Omega_{c}^{\prime }$	$\Omega_{h}$(10)	$\Omega_{h}$(75)	${\tilde{\Omega }}_{h}$
50 (5)	50.5	298.64	54.45	1.01	5.97	3.76
50 (0)	6.75	49.77	22.34	0.135	0.99	3.27
30 (3)	9.72	71.67	8.69	0.324	2.39	1.51
30 (0)	2.43	17.92	4.48	0.081	0.60	1.41
10 (1)	0.54	3.98	0.63	0.054	0.39	0.58
10 (0)	0.27	1.8	0.54	0.027	0.18	0.54

## Abbreviations

The following abbreviations are used in this manuscript:

AUROC	Area under the curve of the receiver operating characteristic
DoS	Denial-of-service
DSR	Dynamic source routing
DT	Decision tree
FPR	False positive rate
FSM	Finite state machine
KNN	k-nearest neighbor
LSTM	Long short term memory
MANET	Mobile ad hoc networks
ML	Machine learning
RNN	Recurrent neural network
RREP	Route reply
RREQ	Route request
RTR	Request-to-receive
SVM	Support vector machine
TPR	True positive rate
TTL	Time to live
